# The activity of platelet enzymes and subpopulation composition of monocytes in schizophrenia

**DOI:** 10.1192/j.eurpsy.2024.1431

**Published:** 2024-08-27

**Authors:** T. Prokhorova, I. Boksha, Z. Sarmanova, O. Savushkina, E. Tereshkina

**Affiliations:** FSBSI “MENTAL HEALTH RESEARCH CENTRE”, Moscow, Russian Federation

## Abstract

**Introduction:**

The studies on various groups of patients with schizophrenia revealed impairments in immune system, glutamatergic, and antioxidant systems contributing substantially in schizophrenia pathogenesis.

**Objectives:**

To search for links between the activities of platelet enzymes involved in glutamate and glutathione metabolism and monocytes’ subpopulation compositions in patients with schizophrenia and to identify possible correlations of the biomarkers with clinical data. Research objectives: determination of subpopulation ratio of monocytes; measurement of the activity levels of glutamate dehydrogenase (GDH), phosphate-activated glutaminase (PAG), glutathione reductase (GR) and glutathione S-transferase (GST) in blood platelets; search for correlations between these parameters and the scores by psychometric scales.

**Methods:**

The study included 36 women aged 16-45 years with acute schizophrenia hospitalized in the Mental Health Research Centre with their current condition assessed as depressive-delusional. The control group consisted of 17 women 18-45 years old without somatic or mental pathology. GDH, PAG, GR and GST activities were measured by spectrophotometric methods, and numbers of monocyte subpopulations - “classical”, “intermediate”, “non-classical” - by flow cytometry. The Hamilton Depression Rating Scale (HAMD-17) was used to assess depression severity. The data was processed using the Statistica 8.0 software.

**Results:**

The detected changes in monocyte subpopulations’ composition towards the increase in the proportion of cells having a pro-inflammatory phenotype (CD14++CD16+ “intermediate”) indicated the activation of inflammatory reactions. Also, the activities of platelet enzymes of glutathione metabolism (GR and GST) were significantly decreased (p<0.05). Moreover, GDH and GST activities significantly correlated with the scores by HAMD-17 (r=0.40, p=0.022 and r=0.45, p=0.030, respectively). The results indicate the presence of pathological inflammatory process, the decrease in activities of glutathione antioxidant metabolism enzymes and a link to glutamate metabolism involvement (GDH) in the studied patient group.

**Conclusions:**

The identified redistribution in the monocyte subpopulations’ composition and decrease in the activity of enzymes involved in glutamate metabolism and antioxidant system indicate the involvement of the immune, glutamate and antioxidant systems in the pathogenesis of schizophrenia and may reflect a functional interaction between these systems.

**Disclosure of Interest:**

None Declared

